# Developments in Blood-Brain Barrier Penetrance and Drug Repurposing for Improved Treatment of Glioblastoma

**DOI:** 10.3389/fonc.2018.00462

**Published:** 2018-10-23

**Authors:** Bryan G. Harder, Mylan R. Blomquist, Junwen Wang, Anthony J. Kim, Graeme F. Woodworth, Jeffrey A. Winkles, Joseph C. Loftus, Nhan L. Tran

**Affiliations:** ^1^Departments of Cancer Biology and Neurosurgery, Mayo Clinic Arizona, Scottsdale, AZ, United States; ^2^Department of Health Sciences Research, Center for Individualized Medicine, Mayo Clinic Arizona, Scottsdale, AZ, United States; ^3^Department of Neurosurgery, University of Maryland School of Medicine, Baltimore, MD, United States; ^4^Department of Surgery, University of Maryland School of Medicine, Baltimore, MD, United States; ^5^Department of Biochemistry and Molecular Biology, Mayo Clinic Arizona, Scottsdale, AZ, United States

**Keywords:** GBM, glioblastoma, Blood-brain barrier, repurposed drugs, recurrent GBM, pharmacotherapy

## Abstract

Glioblastoma (GBM) is one of the most common, deadly, and difficult-to-treat adult brain tumors. Surgical removal of the tumor, followed by radiotherapy (RT) and temozolomide (TMZ) administration, is the current treatment modality, but this regimen only modestly improves overall patient survival. Invasion of cells into the surrounding healthy brain tissue prevents complete surgical resection and complicates treatment strategies with the goal of preserving neurological function. Despite significant efforts to increase our understanding of GBM, there have been relatively few therapeutic advances since 2005 and even fewer treatments designed to effectively treat recurrent tumors that are resistant to therapy. Thus, while there is a pressing need to move new treatments into the clinic, emerging evidence suggests that key features unique to GBM location and biology, the blood-brain barrier (BBB) and intratumoral molecular heterogeneity, respectively, stand as critical unresolved hurdles to effective therapy. Notably, genomic analyses of GBM tissues has led to the identification of numerous gene alterations that govern cell growth, invasion and survival signaling pathways; however, the drugs that show pre-clinical potential against signaling pathways mediated by these gene alterations cannot achieve effective concentrations at the tumor site. As a result, identifying BBB-penetrating drugs and utilizing new and safer methods to enhance drug delivery past the BBB has become an area of intensive research. Repurposing and combining FDA-approved drugs with evidence of penetration into the central nervous system (CNS) has also seen new interest for the treatment of both primary and recurrent GBM. In this review, we discuss emerging methods to strategically enhance drug delivery to GBM and repurpose currently-approved and previously-studied drugs using rational combination strategies.

## Introduction

Glioblastoma (GBM) is the most common and deadly primary brain tumor in adults. The World Health Organization (WHO) classifies GBM as a grade IV astrocytoma, which carries a dismal prognosis, resulting in an ~30% survival rate over 1 year, with ~3–5% of patients surviving beyond 5 years (1, 2). Upon diagnosis, patients undergo maximal safe surgical resection to remove the bulk of the tumor, followed by radiotherapy (RT) and concomitant oral chemotherapy using the DNA-alkylating agent temozolomide (TMZ) (3, 4). Unfortunately, GBM cells are invariably left behind following surgery due to their highly invasive nature. The residual, invasive tumor cells contribute to near universal tumor recurrence (5–8). There are few effective treatment options for patients with recurrent GBM that prolong lifespan and the median survival rate remains at ~8 months (9). Numerous clinical trials aimed at treating recurrent GBM have failed to improve survival due to unexpected toxicity or ineffectiveness related to limited efficacy and/or targeted action against specific signaling networks that drive tumor recurrence.

Chromosomal, mutational, copy number variation, gene-expression, and proteomic analyses have provided a well-defined characterization of the molecular landscape of primary GBM tumors, but much less is known about recurrent tumors (10, 11). Despite the current state of knowledge regarding GBM biology, little progress has been made in the form of new pharmacological agents as stand-alone or adjuvant therapies. GBM is notoriously heterogeneous which limits the therapeutic value of agents that strictly target a single aspect of the disease within the broad pool of redundant pathways and potential targets (12, 13). It is the pronounced molecular and cellular heterogeneity present within these tumors that creates a substantial therapeutic challenge. This biological feature creates the potential for therapy-resistant subpopulations of GBM cells within the tumor to survive and evolve when exposed to single agent therapies and lead to recurrent tumors from these resistant clones which are refractory to available treatments.

Even as new information becomes available regarding recurrent GBM biology, multiple therapeutic delivery challenges remain, and these must be overcome to effectively treat recurrent GBM. As such, the majority of approved cancer drugs do not readily cross the blood-brain barrier (BBB), significantly limiting the options for GBM treatment. Therefore, exploring new avenues to enhance drug delivery into the brain to treat GBM are currently underway. Some techniques include convection-enhanced delivery, high-intensity focused ultrasound, delivery of drug-packaged nanoparticles, and antagonism of efflux pumps (14). In addition to improving the delivery of drugs with poor BBB permeability, there is a greater focus on the development of drugs that are predicted to cross the BBB, as well as repurposed drugs that are known to cross the BBB.

## Factors limiting pharmacotherapy for GBM

### Blood-brain barrier (BBB)

The BBB is formed by endothelial cells connected by tight junctions and functions to protect the brain from infectious agents and environmental neurotoxicants (15). Astrocytes, pericytes, and perivascular macrophages also contribute to the structure of the BBB, and as maturation occurs, astrocytic end feet line the perivascular space, and pericytes and perivascular macrophages line the basal lamina of the endothelial cells in order to help maintain rigidity (16, 17). Although some molecules are able to passively cross through the endothelial cell monolayer, the expression of efflux pumps, such as P-glycoprotein, actively transports them back into the blood. Because of this efflux system, many drugs display a high brain efflux index (BEI), preventing most cancer drugs from entering normal brain tissue, rendering clinically relevant concentrations of precision-targeted therapeutics unattainable (18). Certain physiochemical properties such as molecular weight, lipophilicity, and charge affect a molecule's ability to permeate the BBB and identification of efficacious drugs that are indicated for the treatment of GBM which meet all of these requirements is difficult. Thus, *in silico* predictive modeling systems have been put in place to examine whether certain pharmacophores have the potential to cross the BBB (19, 20). Despite selecting for drugs that exhibit ideal features for BBB permeability, other factors such as the electrostatically charged and anisotropic brain extracellular space (ECS), which contains a dense network of extracellular matrix (ECM) proteins which can bind drugs and inhibit tissue penetration (21, 22), and the glymphatic system (GLS), which is a conduit for the clearance of many therapeutics from the brain parenchyma into the lymphatic system and blood, are additional barriers that preclude effective drug delivery to and retention in the brain (23–25).

### Drug distribution

For molecules that bypass the BBB, additional challenges are met once at the site of the tumor. GBM displays an invasive phenotype at the rim of the tumor, where cells invade into the brain parenchyma; however, the bulk of the tumor, primary or recurrent, has a high degree of mitotic activity, forming a densely-packed region of cancer cells. Drug distribution is severely limited within the bulk tumor, due to the absence of a functional vascular network. An increased interstitial fluid pressure (IFP) between cells and a limited blood supply results in varied concentrations of chemotherapy being exposed to different regions of the tumor (26, 27). It has been postulated that treatment can drive clonal evolution, either through the selection of clones with drug-resistant molecular profiles or drug-induced genomic alterations, driven by sub-lethal doses of drug (28).

### Tumor hypoxia

Without neovascularization occurring to meet the nutritional demands or bring oxygen toward the center of the tumor, GBM cells use certain mechanisms to survive these harsh conditions. Most notably, as is the case for many solid tumors, ATP production through glycolysis occurs in both oxygenated and oxygen-depleted (hypoxic) conditions. Tumor acidity, potentially due to enhanced glycolysis, has been shown to alter uptake of certain drugs into tumor cells (29). Drugs are able to pass through the membrane more easily when in the ionized form, but are protonated at low pH, making cellular uptake less efficient.

Hypoxia is frequently observed in certain regions of tumors. Hypoxic cells divide slowly and have greater energetic demands, but maintain viability through other cell-survival mechanisms. The transcription factor hypoxia inducible factor 1 (HIF-1) induces a transcriptional program which up-regulates factors that contribute to angiogenesis and the activation of macroautophagy (autophagy) (30). These mechanisms of cell survival confer a malignant phenotype and are attractive targets for GBM treatment (31, 32). The monoclonal antibody bevacizumab (tradename: Avastin) targets the angiogenic protein vascular endothelial growth factor-A (VEGF-A), and suppresses the formation of nascent vasculature. Bevacizumab has been approved for the treatment of recurrent GBM, but does not have any impact on overall survival (33, 34). Autophagy was initially described as a mechanism of cell death, but new information has revealed this is a stress-response pathway that restores the cell's energy balance when nutrients (or oxygen) are limited. Thus, it has been shown that regions of tumors where autophagy is high often co-localize with regions of hypoxia, and autophagy can promote tumorigenesis (35).

### Glioma stem-like cells (GSCs)

The glioma stem-like cell (GSC) subpopulation has recently been associated with invasion and chemoresistance, which is thought to give rise to recurrent tumors. GSC interaction with the tumor microenvironment and the ability to self-renew has been shown to promote survival and has made these cells extremely difficult to target with chemotherapeutics (36). Importantly, GCSs are located in both hypoxic and highly vascularized regions, surrounded by microglial cells which influence the survival and stem-like state of GSCs (37, 38). The underlying molecular biology regarding the origin of GSCs is still a major research interest; however, ongoing studies are underway to identify the transcriptional programs that endow these GCSs with highly invasive or chemoresistant properties.

## Approaches to mitigate the BBB for drug delivery

Although the blood vessels that supply the tumor core are commonly incompletely formed and leaky, especially as the histological grade of the tumor progresses, the components of a healthy BBB are still present in the invasive regions of most GBM tumors and low grade gliomas (15). Even if the core of the tumor is sustained by abnormal vessels with a degree of permeability to drugs, the cells that inevitably migrate away from the core of the tumor and establish secondary tumors in distant locations within the brain are smaller and supplied by normal brain vasculature and thus remain impenetrable to drugs.

Molecules can enter the CNS via free diffusion through the BBB, which is restricted to lipophilic molecules of < 400 Da in size. Larger molecules necessary for brain function cross the BBB via active transport by pumps located on the apical endothelial surface (carrier-mediated transport, CMT) or through the endocytic process of receptor-mediated transport (RMT) (39). Although there are numerous clinical trials using systemic and directly added interstitial therapeutics aimed at disrupting or bypassing the BBB, progress remains hindered by concerns about efficacy and safety of combinations of BBB penetrating methods with chemotherapeutics for GBM. The methods detailed here each carry risks and benefits that should be critically evaluated for effective delivery of drugs without compromise of healthy brain parenchyma. The strengths and limitations of each strategy is summarizied in Table 1.

**Table 1:** Strategies to improve BBB penetration for enhanced drug delivery.

**Strategy**	**Pros**	**Cons**
Convection-enhanced delivery	Enhanced distribution Drug combination delivery	Invasive Not targeted Expensive
Focused ultrasound	Targeted Non-invasive	Expensive
Vasoactive peptides	Transient Non-invasive	Poor clinical efficacy
Pharmacological disruption	Transient	Short half-life of antagonists Conflicting clinical trial results
Nanoparticles	Targeted Controlled release	Clinical efficacy not demonstrated
Osmotic agents	Transient	Invasive Non-specific
Peptide masking	Targeted Non-invasive	Low efficiency

*BBB, blood-brain barrier*.

### Convection-enhanced delivery—bypassing the bbb

The first studies of convection-enhanced delivery (CED) took place in the early 1990s at the National Institute of Neurological Disorders, where CED was found to be a reliable method for delivering molecules directly into the brain with varying physical properties (40). CED directly bypasses the BBB, relying on bulk flow to move both solutes and water along a pressure gradient. Catheters are inserted into the brain parenchyma and positive pressure is applied, pushing infusates into the extracellular fluid. Through this method, large molecular weight drugs can enter the CNS in a way that does not induce systemic toxicity. CED also allows for control over the spatial distribution of drugs in the brain, unlike drugs delivered systemically. These benefits make CED an attractive possibility for the treatment of GBM. However, early randomized trials with CED and conventionally delivered standard of care (TMZ and radiation therapy) showed that CED did not significantly increase survival, potentially due to “first generation” delivery techniques (41). Tissue damage can also occur in the instance of reflux of infusate, which must be carefully controlled for by adjusting flow rates and the properties of the cannula (42).

### Focused ultrasound with microbubbles—mechanical disruption of the BBB

Focused ultrasound (FUS) can enable localized, selective permeability of the BBB. Initial work on the safety and efficacy of FUS showed that short, pulsed ultrasound waves disrupted the BBB in animal models, but with considerable collateral damage of healthy brain tissue. The introduction of lipid-encased gas-filled microbubbles lowered the frequency and power thresholds required for FUS to disrupt the BBB, allowing for safer treatments. When FUS is applied transcranially to the desired region of the brain, the intravascular microbubbles oscillate in the acoustic field, which produces mechanical forces against the tight junctions of the endothelial cells that line the vessel wall (43). The bubbles may also collapse and swiftly move fluid that is thought to act as a microjet that forms channels between endothelial cells. Notably, the effects of FUS are reversible, generally lasting 4–6 h. Unlike CED, FUS is not invasive, and can be used along with MRI to visualize BBB disruption and target the FUS effects to specific sites (44). FUS does not represent direct administration of the drug past the BBB, but can allow drugs that are administered using traditional methods (e.g., intravenous or intra-arterial) to cross the disrupted BBB at the FUS-treated site. Preclinical models demonstrate that FUS can make the BBB permeable to chemotherapy drugs including TMZ, doxorubicin, methotrexate, and carmustine in rat models of glioma (43). Clincal trials are ongoing in the US, Canada, and South Korea investigating this approach in patients with malignant gliomas.

### Vasoactive peptides—chemical disruption of the BBB

Bradykinin, a nine amino acid peptide, is an inflammatory mediator generated by the kinin-kallikrein system. Bradykinin's physiologic roles include vasodilation, decreasing blood pressure, increasing vascular permeability, and mediating pain sensation. Bradykinin exerts its effects by binding to B2 G-protein coupled receptors, which increases intracellular calcium and activates nitric oxide (NO) synthase. The subsequent increase in NO induces vasodilation and an increase in vascular permeability (14). Studies in the late 80s and early 90s took advantage of this physiology and reported that bradykinin infusion into cerebral vasculature allowed for passage of drugs past the BBB. Sanovich et al. (45) were the first to show that the bradykinin analog RMP-7, also known as labradimil or Cereport, promotes increased BBB permeability. They reported that administration of RMP-7 allowed a tracer molecule to gain access to the CNS via widened gaps in endothelial tight junctions rather than through transcellular mechanisms. This work was extended by the same group who later investigated the systemic effects of RMP-7 in a rodent model of glioma. This study established that RMP-7 exhibits tachyphylaxis with continuous infusion and provided the pharmacokinetic foundation for dosing parameters (46). In a subsequent phase II clinical trial, RMP-7 combined with carboplatin was determined to be no more efficacious than carboplatin alone. RMP-7 also did not change the dose of carboplatin required to reach therapeutic levels and reduce toxicity (47). Given these results, phase III clinical trials with RMP-7 were discontinued.

### Pharmacological disruption of the BBB

Several pharmacological mechanisms of BBB disruption have been uncovered thus far and key agents include adenosine agonists and P-glycoprotein antagonists. Adenosine is an endogenous purine nucleoside that signals through G-protein coupled receptors, including the inhibitory A1 and excitatory A2A receptors. Both neurons and glial cells release adenosine into the CNS, where it serves to regulate the release of neurotransmitters, vasodilation, and local inflammation. Adenosine is thought to allow recruited immune cells to enter the CNS by inducing BBB permeability through the modification of tight junction proteins and cytoskeletal rearrangement. Although adenosine shows promise in preclinical studies (48), its pharmacodynamics may be problematic if administered in the clinic. Adenosine itself has a 10 s half-life and requires adenosine receptors and the surface marker CD73 to be present on the BBB endothelium in sufficient amounts to cause a significant physiological response.

P-glycoprotein is an ATP-dependent drug efflux transporter that comprises the protective role of the BBB. This efflux transporter removes toxicants from endothelial cells, preventing harmful molecules from moving from circulation to the CNS. Fellner et al. (49) showed that the P-glycoprotein inhibitor PSC833 increased taxol accumulation in the mouse brain. Despite success in preclinical investigations, early clinical trials with P-glycoprotein antagonists were disappointing. However, in 2018, de Gooijer et al. found that inhibiting two transport proteins—P-glycoprotein and ABCG2—increased efficacy of TMZ in murine models (50). This study supports the reconsideration of drug efflux pump antagonism as a means of accessing CNS tumors despite earlier negative results.

### Nanoparticles

Recently, nanoparticles of a variety of compositions have been investigated for their ability to carry drugs across the BBB [for a focused review, see Hersh et al. (14)]. They are typically administered intravenously and have varying ability to penetrate the BBB and remain in circulation long enough to have an effect (51). Studies of nanoparticles for drug delivery across the BBB must optimize the combination of drug, stabilizer, and composition of the nanoparticle to maximize the stability in circulation, the mechanism by which the cargo gets past the BBB, and the avoidance of uptake by the mononuclear phagocyte system (MPS).

Polymeric nanoparticles encapsulate drugs and cross the BBB via endocytosis. Several combinations of polymers, stabilizers, and drugs have been investigated so far. A 2018 study by Li et al. highlights the potential for combining both previously established and novel approaches for getting drugs across the BBB. This study used polysorbate-80-stabilized poly (D,L-lactide-co-glycolate) (PLGA) polymeric nanoparticles loaded with paclitaxel paired with FUS in mouse models of glioma. They found that using PLGA nanoparticles and FUS in combination to deliver paclitaxel across the BBB disrupted endothelial cell tight junctions, decreased P-glycoprotein expression, and allowed for greater antitumor efficacy of the paclitaxel (52). Liposomes, like polymeric nanoparticles, can also encapsulate drugs. Liposomes represent an option for both hydrophobic and hydrophilic drug delivery and these drug carriers are relatively easy to prepare and carry little risk of toxicity. However, the MPS readily recognizes and removes liposomes from circulation, so it is necessary for the surface of liposomes to be modified with antibodies targeting RMT proteins (see below), or chemicals that make them smaller and more difficult for the MPS to recognize (51).

In the case of metallic nanoparticles, drugs can be conjugated to the surface, but cannot be contained within the particle itself. One study found that transactivator of transcription (TAT) peptide-modified gold nanoparticles (TAT-Au NP) can cross the BBB and deliver doxorubicin and gadolinium contrast agent to brain tumor tissue in a murine intracranial glioma xenograft model (53).

Unlike artificially synthesized nanoparticles, exosomes represent endogenous cell-derived particles that can potentially be harnessed for drug delivery. They are thought to be more stable than liposomes and they express surface markers for cell-cell communication that make them ideal for manipulation of the RMT system (54).

### Osmotic agents—mannitol/arabinose

Low concentrations of mannitol are already used routinely to decrease intracranial pressure following traumatic brain injuries and in brain tumor patients (55), and this technique has been shown to allow a variety of intra-arterially administered agents to cross the BBB, including small molecule drugs, peptides, and viral vectors (56). In osmotic disruption of the BBB, hypertonic arabinose or mannitol solutions are infused into the carotid artery for 30 s. This infusion of hypertonic solution causes endothelial cells to shrink as they lose water to the temporary osmotic gradient. This shrinkage widens the gaps between endothelial cells. This permeability is compounded by vasodilation, which occurs as water leaves cells and subsequent rising intracellular calcium levels modulate the contraction of the endothelial cell cytoskeleton. It is estimated that osmotic disruption of the BBB causes a 10-fold increase in permeability that lasts ~10 min. Osmotic disruption, although widely applicable, is not selective for specific sites in the brain, introducing the risk of toxicants from the circulatory system gaining access to the CNS. The rebound phenomenon also represents a risk to consider specifically with the use of mannitol in GBM patients. Mannitol can leak through the disrupted BBB in parts of solid tumors and cause a reversal of the osmotic gradient. This rebound phenomenon can increase edema surrounding the tumor and increase intracranial pressure rather than decrease it (57).

### Peptide masking

The underlying principle of peptide masking is to trigger endogenous RMT mechanisms to endocytose cargo into the BBB endothelium. This can be achieved by conjugating drugs with peptides, receptor ligands, or antibodies that initiate RMT pathways. Some examples of receptors on the endothelial surface that are targets for the induction of RMT include transferrin receptor, insulin receptor, low-density lipoprotein receptor (LDLR), diphtheria toxin receptor, and heparin binding epidermal growth factor like growth factor (58). A phase I study of GRN10005, a peptide-drug conjugate that targets LDLR-related protein 1 was found to successfully deliver paclitaxel across the BBB of patients with recurrent glioma (59). Investigators working to design preclinical and clinical studies of peptide masking must evaluate not only the choice of peptide to trigger RMT, but also the choice of peptides that will target the GBM cells themselves. There is a need for peptides that can function both to initiate RMT and target GBM cells once they cross the BBB (58).

## Repurposing drugs with BBB-permeability for GBM treatment

**Figure 1:**
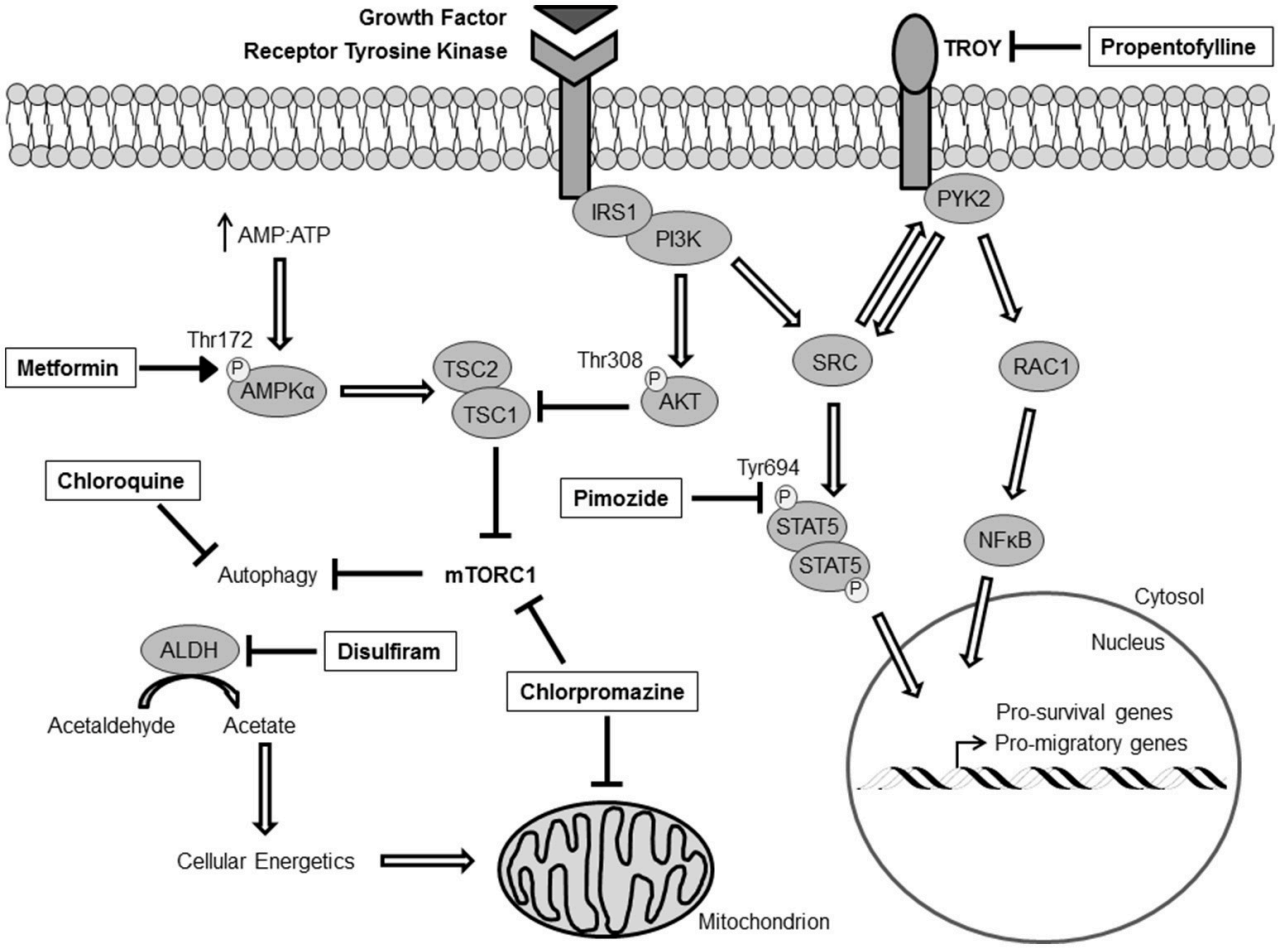
Critical signaling networks in GBM that have druggable targets. All indicated drugs (boxed) exhibit current/former FDA-approval status, established BBB-penetrance, and evidence targeting GBM survival *in vitro* and *in vivo*. IRS1, Insulin receptor substrate 1; PI3K, Phosphatidylinositol-4,5-bisphosphate 3-kinase; PYK2, Protein tyrosine kinase 2; RAC1, Ras-related C3 botulinum toxin substrate 1; NFκB, Nuclear factor kappa-light-chain-enhancer of activated B cells; STAT5, Signal transducer and activator of transcription 5; TSC, Tuberous Sclerosis Complex; AMPK, AMP-activated protein kinase; ALDH, Acetaldehyde dehydrogenase.

Due to the limitations and the side-effects caused by opening the BBB to augment drug delivery, another strategy to make novel treatment options readily available for GBM patients would be to explore FDA-approved drugs with known BBB penetrance and CNS activity. Since the implementation of the Stupp protocol in 2005, the treatment strategy of removing the primary tumor, followed by RT with concomitant TMZ, has not changed. Therefore, a great deal of effort has been placed on finding drugs that enhance the effects of RT and TMZ, but a greater emphasis should be placed on understanding recurrent tumor biology and the pathways that drive survival, proliferation, and invasion, and finding drugs that have current FDA-approval to inhibit these pathways. The following is a partial list of drugs either with current or former FDA-approval for alternate indications that penetrate the BBB, target established and emerging factors that are required for GBM survival, and have strong pre-clinical/clinical evidence for use against GBM. These drugs and their mechanisms of action in GBM cells are summarized in Figure 1.

### Metformin

The biguanidine metformin is indicated for the treatment of Type 2 diabetes. It is orally available and acts by decreasing hepatic glucose production through the activation of AMP-activated protein kinase (AMPK). Activated AMPK (phosphorylated at threonine 172 of the alpha subunit) is a known repressor of mTOR activity through phosphorylation and activation of TSC2 (60). Multiple reports have identified overactive mTOR signaling in GBM, and inhibiting the downstream effects of mTOR is a common therapeutic approach (8, 61). Accordingly, metformin has been shown to sensitize glioma cells and glioma stem cells to TMZ both *in vitro* and *in vivo*, and has been used in a Phase 1 clinical trial for GBM (62, 63). Additionally, targeting both oxidative phosphorylation and glycolysis with metformin and 2-DG synergistically inhibits cellular bioenergetics, resulting in a loss of stemness and viability in GBM tumorspheres and offers a survival benefit in an orthotopic xenograft mouse model (64).

### Propentofylline

Propentofylline (PPF) is a xanthine derivative and a well-established inhibitor of the phosphodiesterases. This activity of PPF in microglial cells reduces the mechanisms that drive inflammation, which has been thought to contribute to vascular dementia and Alzheimer's disease. After extensive testing, results from a Phase III clinical trial reported that PPF did not provide a benefit for people with dementia or Alzheimer's disease, and was subsequently withdrawn from trials in humans, despite a good safety profile and documented brain accumulation. In the context of cancer, PPF was shown to inhibit the pro-tumorigeneic effects of microglia in a rodent model of glioblastoma (65). PPF was found to target TROY, an orphan receptor in the Tumor Necrosis Factor Receptor (TNFR) superfamily, which is highly expressed on microglia and drives microglial migration toward CNS-1 cells (66). A later study also found that glioma cells express high levels of TROY, which confers an invasive and chemoresistant phenotype (67, 68). Indeed, PPF was able to blunt the invasiveness and survival of GBM cells by decreasing TROY expression (69). Despite its effectiveness on suppressing the pro-tumorigenic functions of microglia in the tumor microenvironment and on GBM cells that overexpress TROY directly, the mechanism by which PPF inhibits TROY expression remains unknown.

### Pimozide

There is a significant amount of literature suggesting that antidepressant and antipsychotic drugs should be repurposed for the treatment of GBM because of their established CNS activity (70). Pimozide is an antipsychotic drug of the diphenylbutylpiperidine class that was FDA-approved in 1985. It is currently used for the treatment of psychotic disorders such as tourette's syndrome, schizophrenia, and bi-polar disorder, but more recent data from a drug repurposing screen showed that pimozide had a pronounced effect on prostate cancer and acute myeloid leukemia cells via inhibition of STAT5 signaling (71). A recent report from our group identified overactive STAT5 signaling downstream of the constitutively active EGFR variant III (EGFRvIII), and pimozide treatment was able to decrease the migration and survival of GBM cells alone in a STAT5-dependent manner (72). Additionally, TMZ was shown to be more effective in combination with pimozide. STAT5 was shown to drive the expression of the TNFR family member fibroblast growth factor-inducible 14 (Fn14), a transmembrane protein reported to induce cancer cell invasion and survival, and pimozide was able to decrease the expression of Fn14 in a STAT5-dependent manner. Therefore, additional studies are warranted to observe the effects of pimozide in combination with other anti-cancer therapeutics in tumors that display enhanced STAT5 signaling.

### Disulfiram

Disulfiram is a well-known inhibitor of acetaldehyde dehydrogenase (ALDH) and commonly used to treat chronic alcoholism. Recent data has suggested that disulfiram may be effective against GBM. High ALDH1 expression in GBM has been reported, identifying it as a key factor in maintaining brain tumor stem cell capacity (73). Inhibition of ALDH activity with disulfiram results in perturbations of cellular energetics and thus affects migration and viability of GBM cells (74). Additionally, ALDH expression has been implicated in TMZ resistance; however, there is a report identifying disulfiram as an inhibitor of the DNA repair enzyme MGMT by reducing its protein levels, thereby re-sensitizing GBM cells to alkylating agents and augmenting DNA-damage-induced apoptosis (75, 76).

### Chloroquine

Chloroquine has been an effective anti-malaria drug for decades. It is known to inhibit the life cycle of the malarial parasites belonging to the *Plasmodium* genus; however, resistance to chloroquine has occurred in different regions of the globe, forcing the production of other anti-malarial drugs with different mechanisms of action. Interestingly, chloroquine has emerged as an attractive anti-cancer therapy due to its effect on the inhibition of lysosome-mediated degradation. The inhibition of lysosomal-mediated degradation also affects the late stage of autophagy, inhibiting the completion of autophagic flux and causing a build-up of cellular cargo and debris that is meant to be broken down. This imbalance in proteostasis forces cells to undergo apoptosis, which is why chloroquine has been shown to be an effective adjuvant cancer therapeutic (77). The use of chloroquine in combination with other cancer drugs with distinct mechanisms of action could be beneficial because of the likelihood that autophagy is induced by other anti-cancer drugs as a cell-survival mechanism.

Pre-clinical studies indicate that inhibition of autophagy with chloroquine can sensitize glioma cells to the cytotoxic effects of TMZ (78, 79). This approach has also been tested in the clinic, and hydroxychloroquine was shown to be more effective with radiation therapy and concurrent and adjuvant TMZ (80). Moreover, a randomized, doubled-blinded, placebo-controlled clinical trial with oral-delivered chloroquine added to conventional therapy was conducted in GBM patients. The addition of chloroquine improved mid-term survival (81). These are encouraging results and large-scale trials are needed to definitively determine if chloroquine should be added as an adjuvant therapy for the treatment of GBM. Unfortunately, one of the limitations to using chloroquine in patients is the fact that high concentrations are needed to achieve the desired lysosomotropic effects, which offers considerable toxicity. As a result, derivatives of chloroquine or other autophagy inhibitors with distinct mechanisms of action and enhanced potency could minimize toxicity in patients and have an overall better outcome in combination with radiation therapy or TMZ.

### Chlorpromazine

Chlorpromazine is an antipsychotic medication used primarily to treat schizophrenia and bi-polar disorder. It was the first typical antipsychotic drug discovered in the 1950s and it is still effective today, even with more potent atypical antipsychotics available. A publication from the mid-1990s showed that chlorpromazine, in combination with BCNU, was an extremely effective treatment regimen in a rat orthotopic glioma model (82). The authors attributed the effects of chlorpromazine to the inhibition of calmodulin. More recently, treatment of C6 glioma cells with chlorpromazine caused cell-cycle arrest at the G2/M phase through transcriptional activation of p21(Waf1/Cip1) (83). This transcription appeared to be mediated through the activation of early growth response-1 (EGR-1), which occurred independent of p53. Moreover, chlorpromazine also had an inhibitory effect on PI3K/AKT/mTOR signaling, leading to a form of caspase-independent cell death (84). Lastly, the effect of chlorpromazine was also tested in a model of chemoresistant patient-derived glioma stem cells. It was determined that chlorpromazine inhibited cytochrome c oxidase (CcO, complex IV) activity (85). Previous research from this group also found that the acquisition of chemoresistance coincides with a switch in the expression of CcO subunit 4 isoform 2 (COX4-2) to COX4-1 (86). Taken together, chlorpromazine may be very useful in the clinic against GBM due to its multiple mechanisms of action. Though the targets of chlorpromazine may be non-canonical survival mechanisms for GBM, this fact may call for the use of chlorpromazine as an adjuvant therapy, rather than a specific, front-line therapy.

## Conclusion

There have been numerous promising developments related to drug penetration through the BBB and the identification of existing drugs that may be repurposed for the treatment of GBM. Coordinated efforts to effectively treat GBM and significantly increase patient survival while minimizing the negative impact of these treatments on brain function will be enhanced by technologies that enable controlled penetration of the BBB and multi-modal treatment of this complex, heterogeneous disease.

## Author contributions

BH wrote most of the manuscript and created the figure. MB contributed to the text. JW, AK, GW, JW, JL, and NT edited the manuscript.

### Conflict of interest statement

The authors declare that the research was conducted in the absence of any commercial or financial relationships that could be construed as a potential conflict of interest. The handling Editor declared a shared affiliation, though no other collaboration, with BH, MB, JW, JL, and NT.
